# The pigment characteristics and productivity shifting in high cell density culture of *Monascus anka* mycelia

**DOI:** 10.1186/s12896-015-0183-3

**Published:** 2015-08-13

**Authors:** Gong Chen, Kan Shi, Da Song, Lei Quan, Zhenqiang Wu

**Affiliations:** School of Bioscience and Bioengineering, South China University of Technology, Guangzhou, 510006 China

**Keywords:** *Monascus* mycelia, High cell density culture, Pigment characteristics, Pigment productivity, Feeding medium

## Abstract

**Background:**

*Monascus* mycelia and pigments are promising sources of food and medicine with their potential pharmaceutical values and health-improving functions. Using high cell density fermentation of *Monascus* spp. to achieve higher mycelium and yellow pigment production is worthy to be researched. In this study, the characteristics and productivity shifting of pigments in high cell density culture of *Monascus anka* GIM 3.592 were investigated.

**Results:**

The high yield of *Monascus* mycelia up to 39.77 g/L dry cell weight (DCW), which was achieved by fed-batch fermentation with the feeding medium containing C, N, P and trace elements, was four times higher than that of conventional batch culture. But the total pigment production decreased by 14.6 %, which suggested non-coupled growth. Potential novel yellow pigments accumulated constantly at the late stage of the fed-batch culture, which resulted in a shift in pigment characteristics so that yellow pigments became the dominant pigments. Citrinin production was extremely low and independent of feeding ingredients.

**Conclusions:**

This study provided a suitable fermentation strategy to produce functional *Monascus* mycelia with a high proportion of yellow pigments in high cell density culture. For the first time, it reported the pigment productivity and characteristics shifting in high cell density culture of *Monascus*.

**Electronic supplementary material:**

The online version of this article (doi:10.1186/s12896-015-0183-3) contains supplementary material, which is available to authorized users.

## Background

The genus *Monascus* has been widely utilized in food and medicine fermentation in Eastern Asia for several centuries [1]. Especially, the mycelium of *Monascus* is being developed as functional fat-reducing and lipid-lowing foods [2]. The microbe can synthesize various secondary metabolites with polyketide structure, including *Monascus* pigments, citrinin, lovastatin (monacolin K), γ-aminobutyric acid and dimerumic acid [3, 4].

*Monascus* pigments are a group of azaphilones mixture that mainly consists of three types of color components (yellow, orange and red) [1, 4]. So far, more than fifty *Monascus* pigments have been isolated and identified [3], among which the yellow *Monascus* pigments have been reported for their potential anti-tumor [5], anti-diabetic, anti-oxidative stress [6], anti-inflammatory [7, 8] and anti-obesity [9] bioactivities. The orange *Monascus* pigments also appeared to have anti-cancer effects [10]. It is noteworthy that citrinin, a mycotoxin with hepatotoxicity and nephrotoxicity, is commonly co-produced with *Monascus* pigments [11]. It presents a disadvantage for the use of *Monascus* mycelia and pigments as functional foods and food additives [12].

Many studies have shown that both *Monascus* pigments and citrinin are derived from polyketide [13, 14], and the production and characteristics of pigments can be regulated by the medium compositions [15, 16] and culture conditions [17–19]. What’s more, it had been reported that fed-batch culture with suitable nutrient supply may provide a better strategy for high density cell growth [20, 21]. Studies on the high density fermentation of *Monascus* pigments showed that the highest yield of biomass could be up to 28 g/L dry cell weight (DCW) [22–24].

In this study, high cell density culture of *Monascus anka* GIM 3.592 was conducted by fed-batch fermentation. The pigment characteristics and productivity shifting in high cell density culture of *Monascus anka* GIM 3.592 were examined and the corresponding metabolic rules were studied.

## Results

### Key factors for cell production in high density culture

**Fig. 1:**
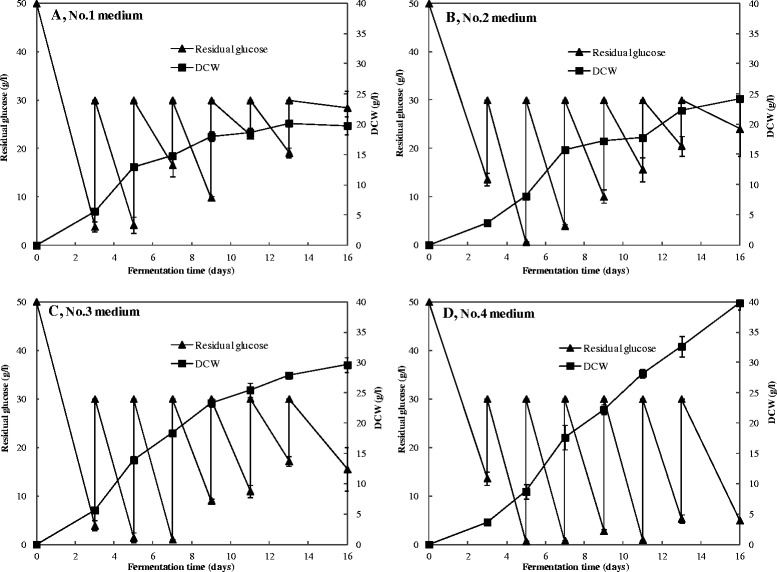
The patterns of residual glucose and DCW with different feeding media in the fed-batch culture. **a** No.1 medium, (**b**) No.2 medium, (**c**) No.3 medium, (**d**) No.4 medium. Error bars represent the standard deviation of duplicate measurements

Four feeding media were used to investigate high cell density growth in fed-batch culture. It’s noteworthy that the cell growth, which was significantly affected by feeding components, increased with the amount of glucose utilization (Fig. 1). Higher glucose consumption rate and cell production rate occurred when nitrogen source were provided as observed for the feeding No.2 medium and No.4 medium (Fig. 1b and d), compared with feeding media No.1 and No.3, respectively; Interestingly, the addition of metal ions in the No.3 medium and No.4 medium led to a remarkable increase of the glucose consumption and cell production (Fig. 1c and d), compared with feeding media No.1 and No.2, respectively. The highest DCW in fed-batch cultures reached 39.77 g/L after 16 days of fermentation when feeding full spectrum of nutrients (No.4 medium), which was four times higher than the maximal DCW in conventional batch culture (9.10 g/L). When feeding the other three media, the final DCW achieved were 19.77 g/L (No.1 medium), 24.24 g/L (No.2 medium) and 29.60 g/L (No.3 medium), respectively.

### Inverse trend between cell productivity and pigment production in high density culture

**Fig. 2:**
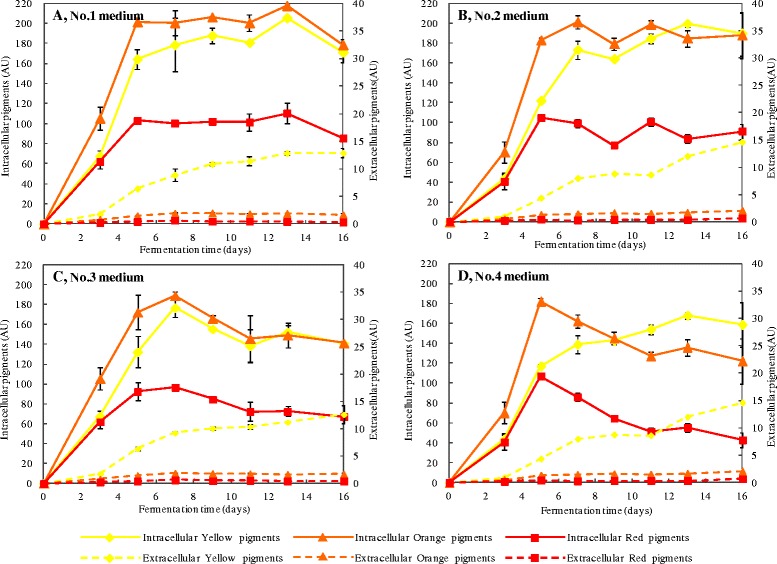
The absorbance of intracellular and extracellular pigments with different feeding media in the fed-batch culture. **a** No.1 medium, (**b**) No.2 medium, (**c**) No.3 medium, (**d**) No.4 medium. Error bars represent the standard deviation of duplicate measurements

The time curves of intracellular and extracellular pigment production in fed-batch cultures with four different feeding media were shown in Fig. 2. For all four feeding media, extracellular pigments were dominated by yellow pigments and reached approximately 14 AU_410_ at the end of the fermentation, which was two times higher than the pigment production in conventional batch fermentation. However, the production of intracellular pigments varied dramatically and depended on the feeding factors. In the initial 5 to 7 days of fermentation, both orange pigments and red pigments accumulated constantly and achieved nearly peak value for all feeding media, and then remained stable for feeding media without metal ions (Fe^2+^, Mn^2+^, Zn^2+^, Mg^2+^) (Fig. 2a and b), or started to decline till the end of fermentation for feeding media with the addition of metal ions (Fig. 2c and d). Intracellular yellow pigments, however, gradually increased till flat to the level of intracellular orange pigments (Fig. 2a, b and c), or even higher than the orange pigments (Fig. 2d), and achieved relatively high values at the end of fermentation.

**Fig. 3:**
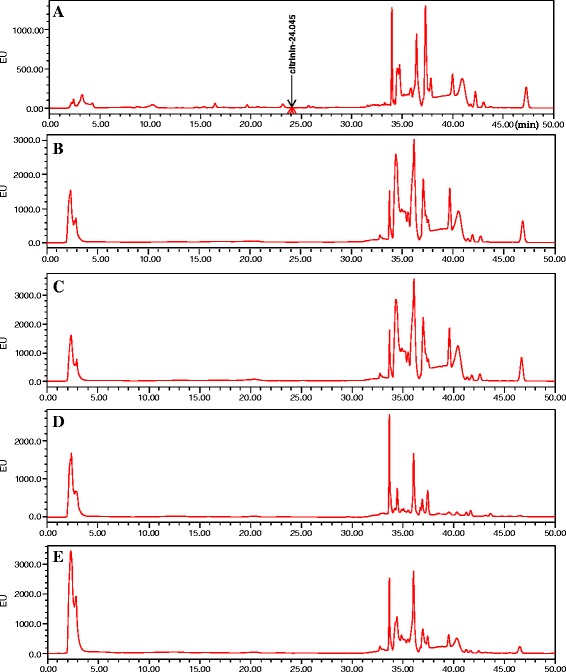
HPLC determination of citrinin with different feeding media in the fed-batch culture. **a** Batch culture (control), (**b**) No.1 medium, (**c**) No.2 medium, (**d**) No.3 medium, (**e**) No.4 medium

Interestingly, we observed an inverse overall trend in cell production and pigment production when feeding with different media. Total pigment production presented a downward trend when feeding media No.1 to No.4 (Fig. 2a–d), which was opposite to the rising trend of DCW (Fig. 1a–d). What’s more, the pigment productivity in all single fed-batch fermentation showed periodic changes that rose early but fell with time, and ended up with a lower value (Table 1). The concentration of citrinin was examined by HPLC after 16 days of fermentation with four different feeding media (Fig. 3). Results showed that no citrinin was detected in the fed-batch culture with any of the four feeding media tested, whereas the conventional batch fermentation produced trace amount of citrinin (<10 μg/L) (Table 1).

**Table 1:** Cell yield and pigment productivity with different feeding media in the fed-batch culture

Fed-batch medium	Culture period (days)	Total glucose consumption (g/L)	DCW (g/L)	Total pigments productivity (AU*g^−1^*d^−1^)^a^	Citrinin (μg/L)
control	6^b^	49.31 ± 0.04	9.11 ± 0.68	7.33 ± 0.43	<10
No.1	16	125.14 ± 3.37	19.77 ± 1.53	1.43 ± 0.16	0
No.2	16	141.80 ± 5.71	24.24 ± 0.74	1.25 ± 0.18	0
No.3	16	171.09 ± 4.50	29.60 ± 1.18	0.77 ± 0.02	0
No.4	16	200.97 ± 0.27	39.77 ± 1.06	0.54 ± 0.01	0

^a^the production of total *Monascus* pigments in per DCW every day

^b^the best fermentation period of conventional batch culture

### Shift in characteristics of *Monascus* pigments in high density culture

**Fig. 4:**
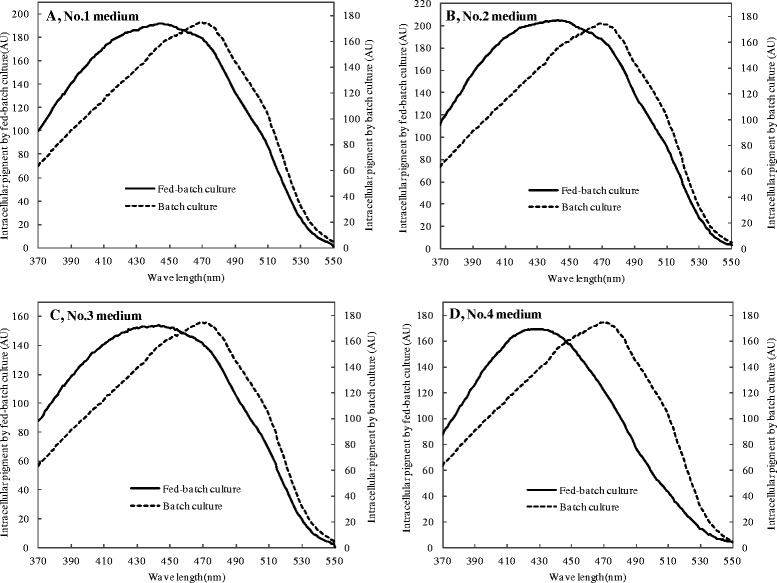
The visual spectra of intracellular pigments with different feeding media in the fed-batch culture. **a** No.1 medium, (**b**) No.2 medium, (**c**) No.3 medium, (**d**) No.4 medium

**Fig. 5:**
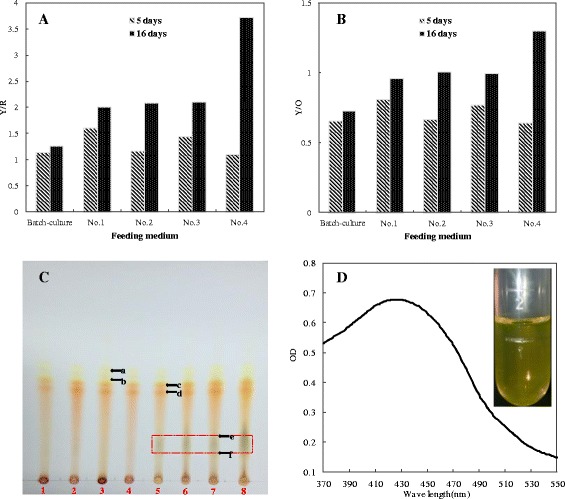
The variation of tone and TLC analysis with different feeding media in the fed-batch culture. **a** The rate of intracellular yellow pigments to red pigments (Y/R). **b** The rate of intracellular yellow pigments to orange pigments (Y/O). **c** TLC atlas in visible light. Lane 1-4: intracellular samples with four different feeding media (from No.1 to No.4) in the 5^th^ days; Lane 5-8: intracellular samples with four different feeding media (from No.1 to No.4) in the 16^th^ days; a and b presented the two yellow pigments at R_*f*_ values of 0.75 and 0.71, c and d presented the two orange pigments at R_*f*_ values of 0.67 and 0.63. **d** The scanning spectrum of the novel yellow substance

The visual spectra of intracellular pigments were shown in Fig. 4. After 6 days of fermentation in conventional batch culture, the spectra of intracellular pigments exhibited an absorbance peak at approximately 470 nm, which was the characteristic absorbance peak of orange pigments [25]. However, the spectra of intracellular pigments after 16 days of fermentation with four different feeding media in fed-batch culture shifted to an absorption peak at approximately 410 nm, which represented the characteristic absorbance peak of yellow pigments [25]. As a result of the constant accumulation of intracellular yellow pigments in the fed-batch fermentation, the yellow tone, as shown by the rate of yellow pigments to red pigments (Y/R) (Fig. 5a) and the rate of intracellular yellow pigments to orange pigments (Y/O) (Fig. 5b), had a significant increase. Intracellular pigments were therefore dominated by the yellow pigments and presented a color of yellow. TLC was then carried out to further characterize the intracellular pigments produced in the fed-batch culture. No significant difference was observed between the intracellular pigments samples on the 5^th^ days of fermentation and those on the 16^th^ days of fermentation, except that the latter samples exhibited a darker band with R_*f*_ values ranging from 0.39 to 0.19 (Fig. 5c, e to f). When extracted by 70 % (*V/V*) ethanol aqueous solution (pH = 2), the pigments extract (R_*f*_ 0.39 to 0.19) displayed a distinct color of yellow, with a single peak of spectrum at approximately 430 nm (Fig. 5d). These pigments may represent a new type of yellow pigments which warrants further investigation.

## Discussion

*Monascus* pigments, as a mixture containing many kinds of colored azaphilone compounds [1], are usually evaluated by their integrated color characteristics [25]. Therefore, we focused more on the variety of integrated color characteristics under high cell density culture, and high yield of *Monascus* mycelia with a high proportion of yellow pigments was achieved by fed-batch fermentation in this study. It has been reported that the accumulation of pigments, as secondary metabolites, is not proportional to cell growth in the fermentation of *Monascus* [22, 26]. The data in our research showed similar results that the DCW constantly increased along with glucose consumption and could reach a high value in fed-batch culture (Fig. 1), whereas production of the pigments increased slowly and even declined in the late stage (Fig. 2), which resulted in low pigment productivity (Table 1). Moreover, no citrinin was accumulated in the process of fed-batch fermentation (Fig. 3 and Table 1), which may be a result from the low final pH (about 2.0, see Additional file 1: Figure S1) in the high cell density culture. It has been reported that nearly no citrinin was biosynthesized at an extremely low final pH [11], and many researches showed that the expression of some biosynthetic gene of secondary metabolites in fungi was affected by the ambient pH [27, 28], i.e. aflatoxin in *Aspergillus Niger* fermentation, sterigmatocystin in *Chaetomium* spp. fermentation and penicillin in *A. nidulans* fermentation [29]. According to the molecular structures of pigments, it has been speculated that *Monascus* pigments are derived from polyketide and fatty acid, the biosynthesis of which share the same precursors and cofactors such as acetyl-CoA, malonyl-CoA, NADH and NADPH [30]. It has reported that a decline in the TCA cycle could lead to an accumulation of acetyl-CoA that is essential for the biosynthesis of polyketide [31]. Therefore, it was possible that the metabolism of polyketide to synthetic pigments and citrinin was inhibited and might be transformed into other pathways during the high cell density fermentation of *Monascus anka*.

The medium compositions have always been suggested to be the key factor for cell growth and pigments metabolism. It has been reported that zinc functions as a catalyst that could promote the more complete destruction of glucose to supply more efficient energy and carbon sources for cell growth [32]. Nitrogen and phosphorus are the major element of cell membranes and nucleic acid, therefore feeding nitrogen and phosphorus sources may also facilitate cell growth. Compared to the “substrate-limited” production of most primary metabolites, secondary metabolism production is usually an “enzyme-limited” process [33]. Higher pigment production was reported in the presence of a combination of Fe^2+^, Zn^2+^ and Mn^2+^ in the batch culture, which was not due to the induction or stabilization of pigment-forming enzymes, but due to increased pigments synthase(s) action; while high concentration of phosphate and MgSO_4_ had an inhibiting effect on the synthesis of *Monascus* pigments [34, 35].

Our data showed that the addition of feeding media with carbon and nitrogen sources resulted in a remarkable increase of DCW (Fig. 1a and b), but no obvious improvement in pigment production (Fig. 2a and b), compared to conventional batch culture. Moreover, the DCW was three to four times higher than that of conventional batch culture when trace elements were fed in the fed-batch culture (Fig. 1c and d), while the total pigment production decreased by 9.0 to 14.6 % (Fig. 2c and d), which may be partly due to the strong negative-regulating effect of high concentration of MgSO_4_ on the action of pigments synthase(s). These findings suggested that feeding carbon and nitrogen sources only played a role in facilitating cell growth, while the trace elements were key factors to improve the cell growth and control pigment synthesis in the high density culture.

We found that the pigment production exhibited almost no decrease from the 6^th^ day to the 16^th^ day of fermentation without feeding ingredients (see Additional file 2: Figure S2). One potential explanation for the decline of intracellular orange pigments and red pigments in the fed-batch culture with feeding No.3 and No.4 medium (Fig. 2c and d) was the transformation of orange pigments to other pigments rather than pigment degradation. It has been widely accepted that orange pigments are synthesized first and the yellow pigments and red pigments are derived from the orange ones [36–38]. According to the molecular structure, it is possible that yellow pigments were formed by hydrogenation of orange pigments [39, 40]. It has been reported that low pH is beneficial for the accumulation of yellow and orange pigments, whereas a relatively high pH and the existence of amino nitrogen source favors the formation of the red pigment [41]. In this study, all the final pH in the fed-batch culture reached about 2.0 (see Additional file 1: Figure S1), which might inhibit the transformation of intracellular orange pigments to red pigments, but facilitate the accumulation of yellow pigments. What’s more, the data showed that the extracellular pigments in the fed-batch culture were dominated by yellow pigments, which could reach almost two times higher than the pigment production in the conventional batch fermentation. The intracellular yellow pigments were gradually increased until flat to orange pigments or even higher than the orange ones in the fed-batch culture (Fig. 2) and the maximum absorption peak of intracellular pigments had a gradually shift to 410 nm compared with the conventional batch culture (Fig. 4), which resulted in a significant improvement of yellow tone (Y/R) at the end of fermentation (Fig. 5a). Moreover, the rate of intracellular yellow pigments to orange pigments (Y/O) was also increased along with fermentation process (Fig. 5b). In summary, the characteristic of *Monascus* pigments had an obvious variation with a high proportion of yellow pigments achieved in the high density fermentation.

Results from TLC showed that the production of two major yellow pigments (R_*f*_ = 0.71 and 0.75) exhibited no obvious change during the fed-batch fermentation (day 16 vs. day 5), but a darker band with R_*f*_ values ranging from 0.19 to 0.39 appeared on the plate (Fig. 5c), which suggested a novel type of yellow pigments. Thus, the increase in intracellular yellow tone observed in the late stage of fed-batch fermentation could be due to primarily the production of the novel type of yellow pigments. The accumulation of high proportion of the yellow pigments, which probably involves variations in the pigment synthase, might reflect the adaptation of *Monascus* spp*.* to the dynamic changes in the external environment in the high density fermentation. Till now, more than 20 yellow pigments have been identified, i.e. xanthomonasins A-B, monankarins A-F, monascusones A-B, monapurones A-C [4, 13]. Future studies are warranted to extract and characterize the novel yellow pigments detected in this study, and to explore the detailed mechanisms of pigment transformation.

## Conclusions

In conclusion, the characteristics and productivity of pigments in high cell density fermentation were significantly affected by the feeding ingredients. High yield of *Monascus* mycelia with almost no citrinin production were achieved by fed-batch fermentation. The total pigment production decreased at the late stage of fermentation, and yellow pigments became the dominant pigments in both intracellular extract and extracellular broth with an accumulation of the potential novel yellow pigments. Overall, this fermentation strategy provides a suitable method to produce functional *Monascus* mycelia with a high proportion of yellow pigments. Further studies are warranted to develop more efficient ways to improve cell production and higher pigments productivity.

## Methods

### Microorganism and cultivation media

*Monascus anka* GIM 3.592 (deposited in the publicly accessible culture collection GDMCC/GIMCC, Guangdong Culture Collection Centre of Microbiology, China) was maintained on potato dextrose agar (PDA) plates and preserved at 4 °C. A sub-culture was carried out at 30 °C for 7 days every month.

The seed medium contained (g/L): glucose 20, yeast extract 3, peptone 10, KH_2_PO_4_ 4, KCl 0.5, and FeSO_4_•7H_2_O 0.01. Fermentation medium contained (g/L): glucose 50, (NH_4_)_2_SO_4_ 5, KH_2_PO_4_ 4, MgSO_4_•7H_2_O 0.5, KCl 0.5, MnSO_4_•H_2_O 0.03, ZnSO_4_•7H_2_O 0.01 and FeSO_4_•7H_2_O 0.01. Four types of feed media were used in the study, i.e. medium 1–4 (Table 2). No.1 medium contained (g/L): glucose 500; No.2 medium contained (g/L): glucose 500, (NH_4_)_2_SO_4_ 50; No.3 medium contained (g/L): glucose 500, KH_2_PO_4_ 40, MgSO_4_•7H_2_O 5, MnSO_4_•H_2_O 0.3, KCl 5, ZnSO_4_•7H_2_O 0.1 and FeSO_4_•7H_2_O 0.1; No.4 medium contained (g/L): glucose 500, KH_2_PO_4_ 40, MgSO_4_•7H_2_O 5, MnSO_4_•H_2_O 0.3, KCl 5, ZnSO_4_•7H_2_O 0.1, FeSO_4_•7H_2_O 0.1 and (NH_4_)_2_SO_4_ 50. All media were at natural pH.

**Table 2:** Four different feeding media in the fed-batch culture

Fed medium	Medium composition
No.1	Glucose
No.2	Glucose, (NH_4_)_2_SO_4_
No.3	Glucose, KH_2_PO_4_, MgSO_4_•7H_2_O, MnSO_4_•H_2_O, KCl, ZnSO_4_•7H_2_O, FeSO_4_•7H_2_O
No.4	Glucose, KH_2_PO_4_, MgSO_4_•7H_2_O, MnSO_4_•H_2_O, KCl, ZnSO_4_•7H_2_O, FeSO_4_•7H_2_O, (NH_4_)_2_SO_4_

### Culture and fed-batch culture

For inoculums cultivation, a suspension of spores was collected by washing PDA plate with 0.1 % tween80 solution, and then approximately 3 × 10^6^ spores were inoculated into a 250 ml Erlenmeyer flask containing 50 ml seed medium. The flask was shaken at 30 °C with 180 rpm for 30 h. After the seed culture, 2 ml of the seed culture broth were inoculated into 25 ml fermentation medium in 250 ml Erlenmeyer flasks, which were incubated at 30 °C with 180 rpm for 6 days in the batch culture.

Fed-batch cultivation was firstly carried out in the fermentation medium as described above on shaking flask. After 3 days of fermentation, a certain amount of feed medium was added to the flasks to keep the glucose concentration at 30 g/L in the whole fermentation broth, in which two flasks were withdrawn from a total of fourteen flasks to analysis residual glucose concentration, biomass and pigments concentration [24]. Since then, an amount of feed medium was added to the flasks every 2 days to keep 30 g/L the glucose concentration in the whole fermentation broth till the end of the experiment.

### Analysis methods

The 25 ml fermentation broth was filtered under vacuum through 0.8 μm mixed cellulose esters membrane. The filtrate was diluted to determine the extracellular pigments concentration and the residual glucose concentration. The mycelia washed by distilled water three times were collected and soaked in 70 % (*V/V*) ethanol aqueous solution (pH = 2) for 2 h, where the volume of ethanol aqueous solution was kept 25 ml as that of the original fermentation broth. The ethanol extract was filtered to separate the mycelia, and the corresponding ethanol aqueous solution was subjected to intracellular pigments concentration and Thin Layer Chromatography (TLC) analysis. The collected mycelia were dried to a constant weight at 60 °C to determine DCW.

Since *Monascus* pigments are a group of azaphilones mixture with multi-components, the concentration of *Monascus* pigments is difficult to determine by standard High Performance Liquid Chromatography (HPLC) method and usually represented by its corresponding absorbance unit (AU, multiplication of the absorbance with its dilution factor of a sample) at their characteristic wavelength. In this study, the concentration of extracellular and intracellular pigments was measured by UV-2000 spectrophotometer at the specific wavelength i.e. 410, 470, and 510 nm that corresponded to the characteristic absorbance of yellow, orange and red pigments, respectively [11, 24]. The residual glucose in the fermentation broth was quantified by the standard 3, 5-dinitrosalicylic acid method (DNS) with a spectrophotometer.

TLC analysis was performed on the Silica gel 60 F_254_ TLC plate (Merck). All the samples spotted on the TLC plate were quantitative and run with n-hexane/ethyl acetate/petroleum ether (30:17:5) as solvent system [42].

The HPLC method used to determine citrinin concentration was modified from Zheng et al [25]. The chromatographic system consisted of a Waters e2695 Solvent Delivery Pump (Waters, USA) and a RF-2475 Fluorescence Detector (Waters, USA). The mobile phase consisted of eluent A (water: HAC = 100:10) and eluent B (acetonitrile: HAC = 100:10) at a flow rate of 1.000 mL/min, the elution gradient was as follows: 0 min, 80 % A and 20 % B; 28 min, 50 % A and 50 % B; 30 min, 15 % A and 85 % B; 50 min, 15 % A and 85 % B.

## Additional files

Additional file 1: Figure S1. The patterns of pH with different feeding media in the fed-batch culture. (PDF 22 kb) Additional file 2: Figure S2. The pigment production in the 6th day and 16th day of conventional batch fermentation. (PDF 196 kb)
