# Supplementary material for: The pigment characteristics and productivity shifting in high cell density culture of Monascus anka mycelia
Source: BMC Biotechnol. 2015 Aug 13;15:72. doi: 10.1186/s12896-015-0183-3 (PMC4535777; doi:10.1186/s12896-015-0183-3)

Additional file 1

Figure S1. The patterns of pH with different feeding media in the fed-batch culture.

Error bars represent the standard deviation of duplicate measurements.

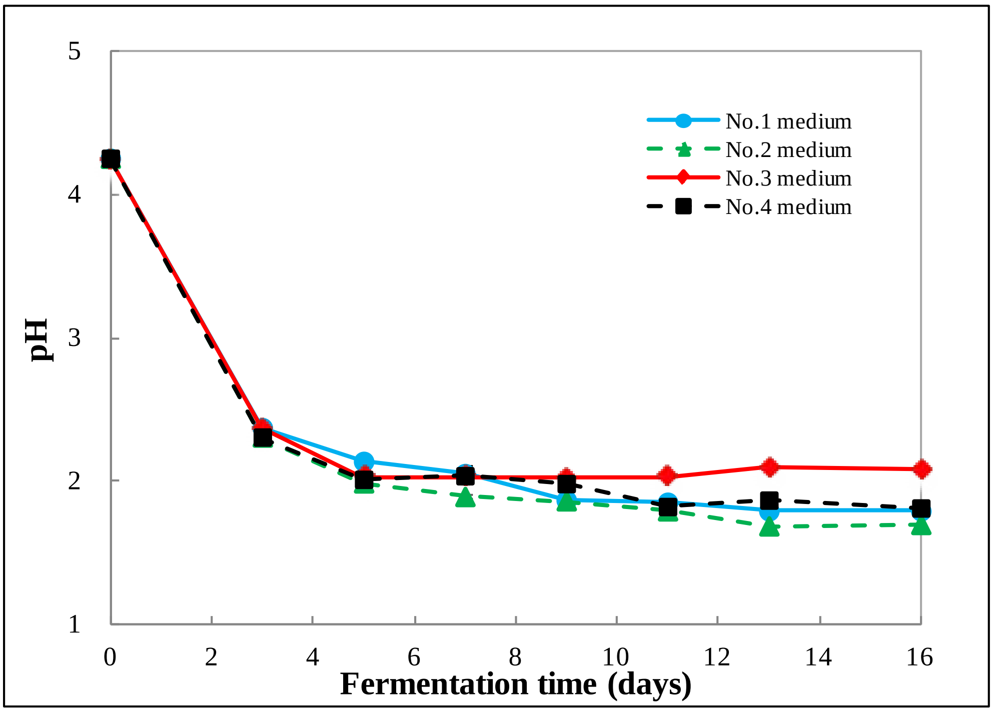

Supplement: Additional file 1: Figure S1. — The patterns of pH with different feeding media in the fed-batch culture. (PDF 22 kb) [file 12896_2015_183_MOESM1_ESM.pdf]
