# Supplementary material for: The pigment characteristics and productivity shifting in high cell density culture of Monascus anka mycelia
Source: BMC Biotechnol. 2015 Aug 13;15:72. doi: 10.1186/s12896-015-0183-3 (PMC4535777; doi:10.1186/s12896-015-0183-3)

Additional file 2

Figure S2. The pigment production in the 6th day and 16th day of conventional batch fermentation.

Both the intracellular pigments and extracellular pigments were shown nearly the same yield in the 6th day and 16th day of conventional batch fermentation with no feeding ingredients.

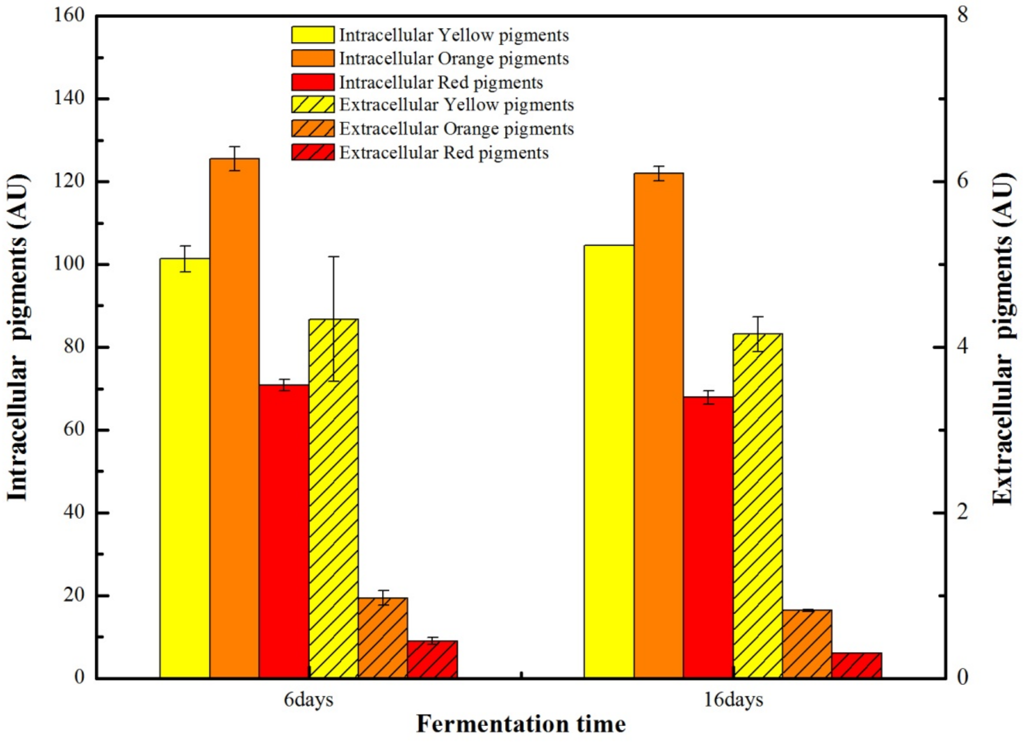

Supplement: Additional file 2: Figure S2. — The pigment production in the 6th day and 16th day of conventional batch fermentation. (PDF 196 kb) [file 12896_2015_183_MOESM2_ESM.pdf]
